# Supplementary material for: Long-Lasting Exendin-4 Fusion Protein Improves Memory Deficits in High-Fat Diet/Streptozotocin-Induced Diabetic Mice
Source: Pharmaceutics. 2020 Feb 16;12(2):159. doi: 10.3390/pharmaceutics12020159 (PMC7076426; doi:10.3390/pharmaceutics12020159)
Supplement: Supplementary file 1 [file pharmaceutics-12-00159-s001.pdf]

pharmaceutics

Supplementary Materials: Long-Lasting Exendin-4 Fusion Protein Improves Memory Deficits in High-Fat Diet/Streptozotocin-Induced Diabetic Mice

Kyung-Ah Park, Zhen Jin, Jong Youl Lee, Hyeong Seok An, Eun Bee Choi, Kyung Eun Kim, Hyun Joo Shin, Eun Ae Jeong, Kyoung Ah Min, Meong Cheol Shin and Gu Seob Roh

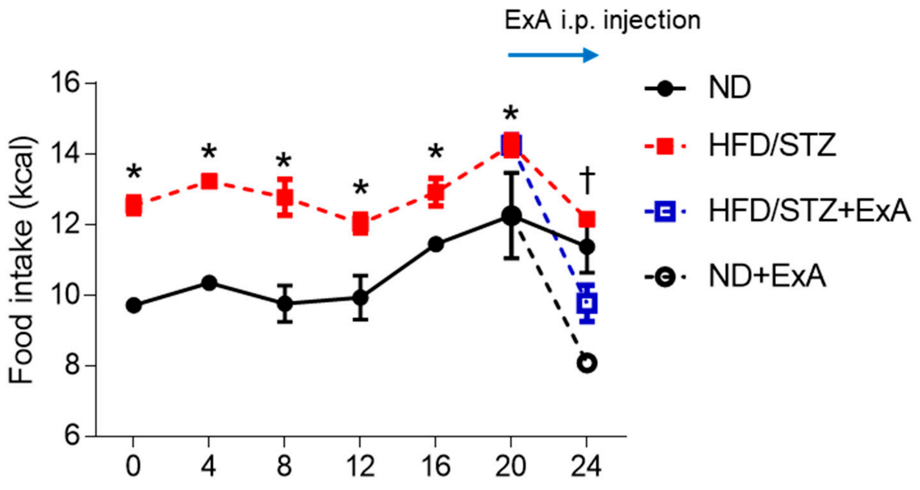

Figure S1. Total calorie intake (kcal) of ND, HFD/STZ, HFD/STZ+ExA, and ND+ExA mice. Total calorie intake (kcal) was calculated from the amount of food intake (g) by each group of mice (ND = 3.1 kcal/g, HFD = 5.24 kcal/g (60 kcal% fat).

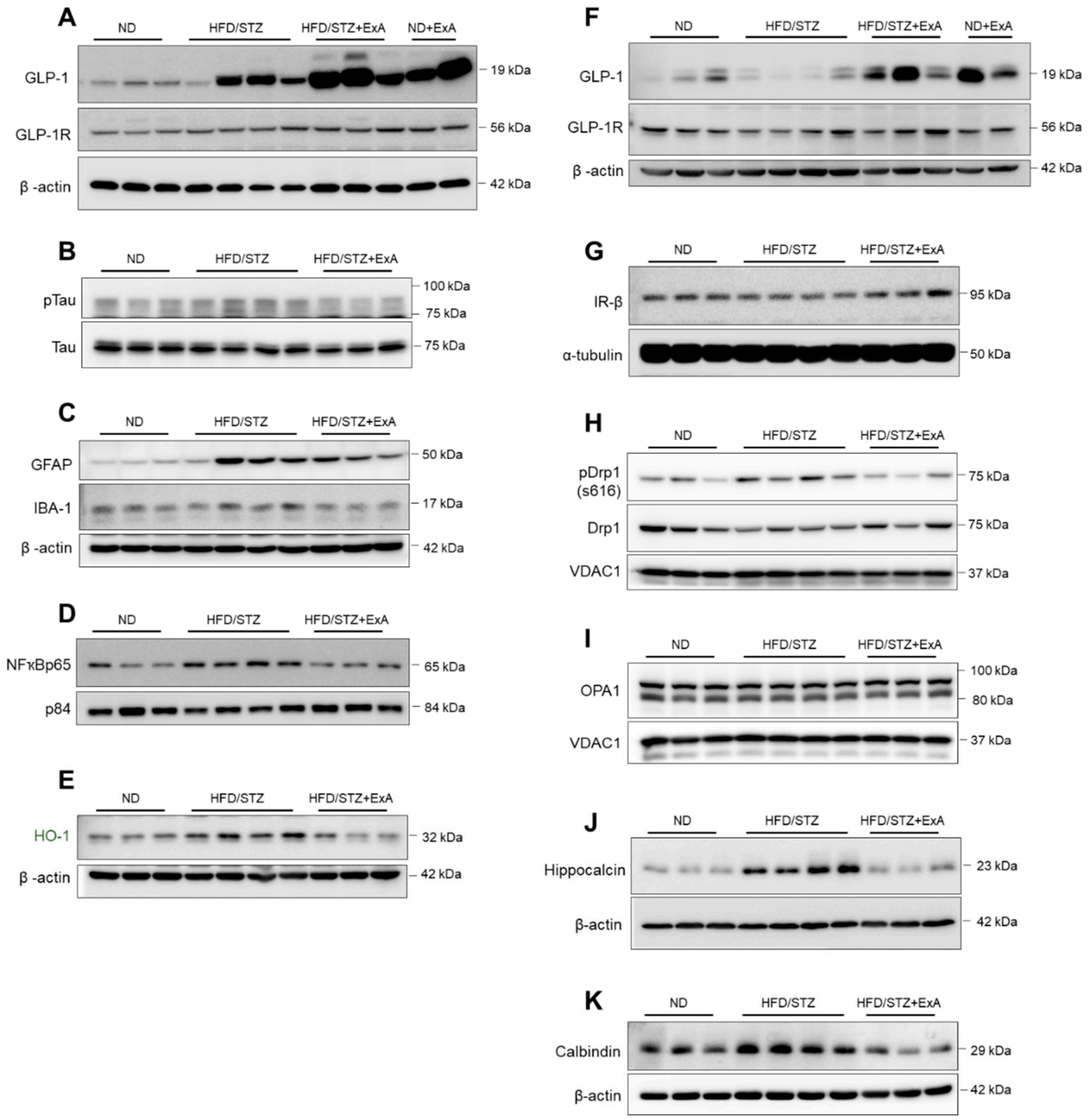

Figure S2. (**A**) Full-length blots of Figure 3F. (**B**) Full length blots of Figure 5E. (**C**) Full length blots of Figure 6A. (**D**) Full length blots of Figure 6C. (**E**) Full length blots of Figure 6D. (**F**) Full length blots of Figure 7A. (**G**) Full length blots of figure 7D. (**H**) Full length blots of Figure 8A. (**I**) Full length blots of Figure 8D. (**J**) Full length blots of Figure 8F. (**K**) Full length blots of Figure 8G.
